# Efficacy of cognitive remediation on activities of daily living in individuals with mild cognitive impairment or early-stage dementia: a systematic review and meta-analysis

**DOI:** 10.1186/s13643-022-02032-0

**Published:** 2022-08-02

**Authors:** Nikki Tulliani, Michelle Bissett, Paul Fahey, Rosalind Bye, Karen P. Y. Liu

**Affiliations:** 1 School of Health Sciences, Western Sydney University, Penrith, NSW Australia; 2 School of Allied Health Sciences, Griffith University, Gold Coast, QLD Australia; 3 Translational Health Research Institute, Western Sydney University, Penrith, NSW 2751 Australia

**Keywords:** Cognitive remediation, Activities of daily living, Ageing, Systematic review, Meta-analysis

## Abstract

**Introduction:**

Instrumental activities of daily living are essential for ageing well and independent living. Little is known about the effectiveness of cognitive remediation on instrumental activities of daily living performance for individuals with mild cognitive impairment or early-stage dementia. The objective of this study was to evaluate the immediate and long-term carryover effects of cognitive remediation on improving or maintaining instrumental activities of daily living performance in older adults with mild cognitive impairment and early-stage dementia.

**Methods:**

Randomized controlled trials published from 2009 to 2022 were identified in OvidSP versions of MEDLINE and Embase, EBSCO versions of CINAHL and PsycINFO, and the Cochrane Central Register of Controlled Trials. A narrative synthesis of the findings was reported on the outcomes of the included studies. Relevant data was extracted and analysed using R software’s ‘metafor’ package with a random effect model with 95% CI.

**Results:**

Thirteen studies, totalling 1414 participants, were identified in the narrative analysis. The results of meta-analysis, inclusive of 11 studies, showed that cognitive remediation elicited a significant improvement in the instrumental activities of daily living performance (*SMD*: 0.17, 95% *CI* 0.03–0.31). There was insufficient evidence of any lasting effect.

**Discussion:**

Cognitive remediation is effective in improving instrumental activities of daily living performance immediately post-intervention in older adults with mild cognitive impairment and early-stage dementia. It appears that individualized interventions with a short duration, such as 10 hours, might be beneficial.

**Systematic review registration:**

PROSPERO CRD42016042364

**Supplementary Information:**

The online version contains supplementary material available at 10.1186/s13643-022-02032-0.

## Background

Mild cognitive impairment (MCI) and dementia are leading causes of disability and dependence in the elderly, constituting a substantial economic burden for public health systems [1, 2]. Globally, dementia alone cost healthcare systems approximately US $594 billion in 2019. It has been predicted that by 2056, dementia spending will increase to US $1.6 trillion [3].

Cognitive decline is prevalent in older adults with MCI and dementia and is associated with a decline in performance of instrumental activities of daily living (IADL) such as completing household chores, shopping, and managing finances [4]. Difficulties with completing IADL may impact on a person’s ability to independently live at home and in the community [5]. Therefore, effective interventions to maintain or improve IADL performance in people with MCI and early-stage dementia are essential to aid successful community-based living and reduce strain on healthcare services.

Cognitive remediation interventions target cognitive decline and can typically be subcategorized into *cognitive training* (CT), *cognitive rehabilitation* (CR), and *cognitive stimulation* (CS). CT uses restorative strategies to improve cognitive performance [6, 7]. CT consists of practising cognitive tasks, focusing on improving or maintaining cognitive functions in one or more cognitive domains [6, 7]. Examples of CT include training in applied memory strategies and mnemonic techniques such as cueing, and method of loci [8] as well as repetitive cognitive exercises targeted cognitive abilities such as spaced retrieval and repeated attention and memory tasks [8]. Unlike CT, CR does not aim to specifically improve cognitive functions. Instead, CR aims to address activity performance problems which arise as a consequence of declining cognition [6, 9]. CR focuses on identifying goals to enhance daily activity performance, providing a tailored intervention for each person. Interventions often include providing compensatory and adaptive strategies at improving performance in specific daily activities. Examples of CR include memory retrieval techniques, activity or environment modification, and errorless learning [7, 9, 10]. CS is another intervention strategy that promotes engagement in daily activities, stimulating general cognitive and social functioning in a nonspecific manner [11]. Examples of CS include activities such as participating in group discussions, reading, playing chess, drawing, and painting. CS aims to boost cognitive reserves and prevent cognitive decline [10, 12].

Cognitive remediation approaches, including CT, CR, and CS, have been shown to be effective methods in reducing the cognitive decline associated with normal ageing and among people with MCI [7, 10]. However, there is lack of evidence on whether these cognitive remediation approaches transfer to everyday living [13, 14]. There has been no systematic review examining the effectiveness of cognitive remediation directly on IADL performance across the continuum of cognitive decline from MCI to early-stage dementia.

## Objective

The objective of this systematic review and meta-analysis is to summarize the available evidence regarding the efficacy of cognitive remediation approaches on the performance of IADL in adults with MCI or early-stage dementia.

## Methods

### Protocol and registration

The methods were published as a protocol before conducting the review [15]. The review was registered on PROSPERO (registration number: CRD42016042364). This review is reported in accordance with the Preferred Reporting Items for Systematic Reviews and Meta-analysis (PRISMA) guidelines [16].

### Search strategy

The following electronic databases were searched: OvidSP versions of MEDLINE and EMBASE, EBSCO versions of CINAHL and PsycINFO, and the Cochrane Central Register of Controlled Trials. The search was tailored to the thesaurus or controlled vocabulary and search syntax of each database and restricted to articles published in English and in peer-reviewed journals. Citation checking was carried out on all included articles and relevant systematic reviews to identify any additional studies missed by the database search. The search was first conducted in March 2019, and an updated search was conducted in June 2022.

The following combinations of keywords were used. All keywords were mapped for ‘index terms’ (e.g. MeSH) and included when relevant.

1. Dementia OR cognitive dysfunction OR Alzheimer disease OR cognition disorders OR MCI OR cognitive impairment no dementia OR memory disorder OR age-associated memory impairment OR age-associated memory disorder OR age-related memory impairment OR aged-related memory disorder OR memory decline OR memory loss OR cognitive decline
2. Cognitive therp* OR cognitive intervention OR cognitive training OR cognitive techniques OR cognitive restoration OR cognitive retraining OR cognitive re-training OR cognitive stimulation OR cognitive rehabilitation OR cognitive remediation OR neurological rehabilitation OR rehabilitation OR mental recall OR mental stimulation OR task training OR occupational therapy OR occupational rehabilitation OR sensory stimulation OR reminiscence therapy OR imagery OR mental imagery OR skill acquisition OR skill retention OR learning OR memory training OR memory encoding OR memory retrieval OR guided imagery OR motor imagery OR visual perception OR visualization OR cues
3. Activities of daily living OR ADL OR IADL OR functional performance OR functional ability OR functional status OR daily task OR daily activities OR complex activities OR task performance OR day-to-day activities
4. Randomized controlled trial OR random*
5. Aged OR older OR elder
6. 1 AND 2 AND 3 AND 4 and 5

The detailed search strategy for MEDLINE is shown as an example in Supplementary Material 1.

### Selection criteria

#### Types of participants

The population included older adults, aged 60 years or above, residing in either the community or within a residential aged care setting, and with a diagnosis of MCI or early-stage dementia as outlined by an established standardized diagnostic criteria such as the following: the National Institute of Neurological and Communicative Disorders and Stroke and the Alzheimer’s Disease and Related Disorders Association criteria [17], Clinical Dementia Rating scale [18], or Petersen’s diagnostic criteria for MCI [19].

#### Types on intervention

Included studies needed to describe a CT-, CR-, or CS-based intervention. No specification was placed on the delivery mode, duration, frequency, or intensity of these interventions.

#### Types of comparators

The comparator provided to the control group could be active controls (for example another intervention) or an inactive approach (for example wait-list control or standard care).

#### Types of outcome measures

The outcome measure was IADL performance. Studies were only included if they reported at least one outcome measure assessing the performance of one or more IADL, provided as a score measured by a valid and reliable scale.

#### Types of studies

This review only included randomized control trials (RCTs).

#### Excluded studies

Articles were excluded if they were as follows: (i) non-intervention studies; (ii) theoretical articles or descriptions of treatment approaches; (iii) review articles; (iv) unpublished studies, abstracts, or dissertations; (v) articles without adequate specification of interventions; (vi) non-peer-reviewed articles and book chapters; and (vii) non-English language articles. Studies which compared two cognitive remediation approaches without a control or standard care were excluded from this review. Multicomponent intervention studies which did not distinguish the contribution of the cognitive remediation component on the effects were also excluded. Studies were excluded from the review if mixed cohorts could not be extracted independently.

Contact was made with corresponding authors for original data if studies included mixed cohorts (including healthy adults, MCI, or dementia, or combining with people younger than 60) and if data for the outcome measure was not reported pre- and post-intervention. Studies were excluded from the meta-analysis if post-intervention data could not be reported, although these studies were included in the narrative analysis of this review.

### Study selection

**Fig. 1:**
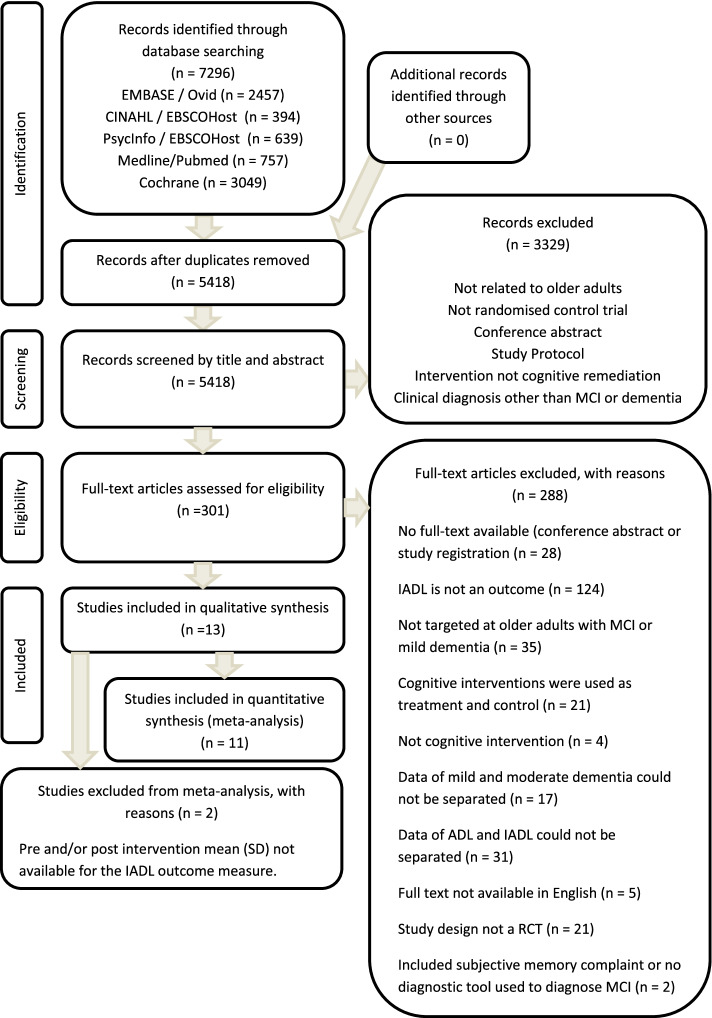
Flow diagram of the study selection process based on the PRISMA guidelines

The study selection process was conducted in accordance with the PRISMA guidelines [16] (Fig. 1). Two independent reviewers (NT and KL) screened the titles and abstracts to determine relevancy to the topic. All papers with study titles and abstracts viewed as relevant by at least one of the two reviewers were retained for full review. Following full review, the reasons for inclusion and exclusion were recorded. Disagreements between the two reviewers were resolved by discussion to reach a consensus.

### Narrative analysis

#### Data extraction

Two independent reviewers (NT and KL) extracted data from the included articles. Where possible, the following information related to the characteristics of the participants, intervention, study design, and results was extracted.

- *Participant characteristics*: (i) Age (mean), (ii) sex, (iii) years of education, (iv) baseline cognitive functioning according to the Mini-Mental Status Examination score, and (v) cognitive diagnostic status
- *Intervention characteristics*: (i) Type and description of cognitive remediation approach, (ii) delivery mode of intervention (individualized or group/independent or facilitated), (iii) duration of training sessions (intensity), and (iv) frequency of sessions per week (dose)
- *Methodological characteristics*: (i) Study design, (ii) study duration, (iii) number of participants, (iv) IADL outcome measure used, (v) duration of follow-up as measured from the end of treatment, (vi) country study took place, and (vii) source of financial support
- *Outcome of Intervention* (*IADL performance*): (i) Baseline IADL score pre-intervention; (ii) IADL score immediately post intervention; (iii) IADL score at follow-up, if applicable; and (iv) reported effect of treatment group on IADL performance immediately following intervention; and (v) reported effect between treatment and control groups immediately following intervention

#### Assessment of risk of bias

Two independent reviewers (NT and KL) assessed the methodological quality of the included studies using the Physiotherapy Evidence Database (PEDro) scale [16]. The PEDro scale consists of 11 items designed to assess the quality and reporting of RCTs [16]. Out of the 11 items, 10 are scored (item 1: eligibility criteria is not scored) [20]. If a study did not report on a particular criterion, the criterion was scored as if it was not met. Based on the criteria, studies were rated as ‘excellent quality’ and low risk if they scored 9–10, good quality and ‘low risk’ if they scored 6–8, fair quality and ‘moderate risk’ if they scored 4–5, or poor quality and ‘high risk’ of bias if they scored 3 or below.

#### Synthesis of results

Summary and descriptive statistics (means and standard deviations [SDs]) were reported for participant and intervention characteristics. A data extraction form was developed and piloted independently by two reviewers (NT and KL) on 10% of the identified studies and modified as required prior to use. Data from all relevant studies was extracted using this form. Disagreements between the two reviewers were resolved by discussion to reach consensus. Corresponding authors were contacted via email for original data where the published data was insufficient for data analysis.

### Meta-analysis

#### Primary analysis

An analysis of combined cognitive remediation approaches (CT, CR, CS) on IADL performance was performed using post-intervention IADL scores (means and SDs) to determine the overall effectiveness of these cognitive interventions. An analysis of the long-term carryover effect at 3–5 months and 6–8 months post-intervention was also conducted. The follow-up period is considered as the period following the initial post-intervention data collection.

#### Subgroup analysis

A subgroup analysis of the different cognitive remediation approaches on IADL performance was performed to determine the intervention with greater effect size. The interventions were categorized into two groups: CR and CT. Studies that used a combined approach were excluded from this analysis. Only one study used CS independently of CR or CT. Therefore, CS was not included in this sub-analysis. A subgroup analysis was also performed based the duration of the intervention. The duration of the interventions was classified into three broad groups: less than 10 h, 10 to 20 h, and 21 to 50 h. Two studies [21, 22] were excluded from this subgroup analysis due to their considerable variation which is duration. A final subgroup analysis examining group intervention and individualized intervention was performed.

If a study compared the effects of cognitive interventions across two treatment groups on the outcome relative to the control, the two treatment groups were combined as described by Higgins, Li [23]. If a study included a treatment group not of interest to this review, it was either used as the control group or not included in the analysis. If a post-intervention score for IADL performance was not available after contact was made with the author, the study was excluded from the analysis.

All analysis was performed using the ‘metafor’ package in R software, where the random effect model with 95% CI was applied [24]. Effect sizes of 0.2, 0.5 and 0.8 represent small, moderate, and large effects, respectively [25].

The statistical heterogeneity of the studies was evaluated using the *I*^2^ statistic. Random effect models were used, as the estimated effects in the included studies were not identical. Meta-analysis with an *I*^2^ between 50 and 90% is considered to have substantial heterogeneity [26]. Publication bias was checked for the primary analysis using the funnel plot asymmetry test. Furthermore, the statistical significance of publication bias was checked using Egger [27] and Begg [28] tests. A *p*-value less than 0.05 was used to determine the presence of publication bias. However, the funnel plot asymmetry test to distinguish chance from real asymmetry has insufficient power when fewer than 10 studies are included [29]. All subgroup analysis included less than 10 studies; therefore, publication bias was not explored.

### Confidence in cumulative evidence

The quality of evidence was assessed using the Grades of Recommendation, Assessment, Development, and Evaluation (GRADE) approach [30] by the first author (NT). Ratings were verified by the senior author (KL). GRADEpro software [31] was used to assess the quality of the evidence in the five domains specified within GRADE: risk of bias, inconsistency of results, indirectness of evidence, imprecision of results, and publication bias [30]. Quality of evidence was rated on a 4-point scale from ‘very low’ (0) to ‘high’ (4). High quality indicates there is a high level of confidence that the true effect lies close to the estimate of effect. Whereas very low quality indicates there is very little confidence that the true effect is close to the estimate of effect, the true effect is likely to be substantially different from the effect estimate [32].

## Results

### Selected articles

A total of 7296 papers were identified. After removal of duplicates, 5418 papers underwent title and abstract review, and 301 were deemed potentially eligible and underwent full-text review. Following full-text review, 13 met the study criteria (Fig. 1). The oldest article is from 2013, and the most recent is from 2022.

### Results of narrative review

#### Participant characteristics

A total of 1414 participants were included from the 13 included studies. The mean age of participants ranged from 71 to 86 years, with 438 males and 976 females. Eight studies included participants with diagnosed MCI [21, 33–39]. A further two studies included participants with probable early stage dementia [33, 40], and the remaining three studies included a combination of participants with either MCI or mild dementia/probable early stage dementia [22, 41, 42] (Table 1).

**Table 1:** Characteristics of included studies and participants

Author, reference	Quality (PEDro score)	Participants								Country	Source of financial support
		Study design	Health condition/diagnosis	Diagnostic criteria	Baseline cognitive status MMSE score Mean (SD)	Sample size (excluding drop outs)	Age (years) Mean (SD)	Sex Male/female	Education Years (SD)		
Barban et al. (2016) [43]	6	RCT (crossover design)	Probable early-stage AD	NINCDS-ADRDA CDR: 1	TG: 23.4 (1.9) CG: 23.4 (1.7)	TG: 42 CG: 39	TG: 76.7 (5.7) CG: 76.9 (5.7)	TG: 13/29 CG: 11/28	TG: 8.8 (3.6) CG: 9.2 (3.7)	Italy, Greece, Norway, and Spain	Co-funded by the European Union in the SOCIABLE project
Belleville et al. (2018) [33]	7	RCT (3-arm design)	MCI	Petersen’s diagnostic criteria for MCI	Not reported	TG1: 40 ^a^TG2: 43 CG: 44	TG: 71.3 (8.5) ^a^TG2: 72.1 (6.7) CG: 73.1 (6.5)	TG1: 20/20 ^a^TG2: 19/24 CG: 18/26	TG1: 14.5 (4.2) ^a^TG2: 14.7 (3.5) CG: 14.8 (3.8)	Canada	Funded by the Canadian Institutes for Health Research
Giuli et al. (2016) [41]	6	RCT (crossover design)	MCI	Petersen’s diagnostic criteria for MCI	TG: 25.7 (1.8) CG: 25.8 (1.9)	TG: 48 CG: 49	TG: 76.0 (6.3) CG: 76.5 (5.7)	TG: 17/31 CG: 19/30	TG: 6.7 (3.8) CG: 5.3 (3.0)	Italy	Funded by the Italian Ministry of Health and the Marche Region
			Probable early-stage (mild) AD	DSM-IV or NINCDS-ADRDA	TG: 20.2 (3.7) CG: 20.3 (3.5)	TG: 48 CG: 47	TG: 76.5 (4.3) CG: 78.7 (5.9)	TG: 19/29 CG: 13/34	TG: 5.9 (4.1) CG: 4.5 (2.3)		
Lam et al. (2015) [21]	6	RCT (4-arm design)	MCI	International Working Group on MCI diagnostic criteria CDR: < 1	TG1: 25.7 (2.4) TG2: 25.2 (2.2) TG3: 25.8 (2.3) CG: 25.6 (2.4)	TG1: 145 ^a^TG2: 132 TG3: 147 ^b^CG: 131	TG1: 74.4 (6.4) ^a^TG2: 76.3 (6.6) TG3: 75.5 (6.7) ^b^CG: 75.4 (6.1)	TG1: 30/115 ^a^TG2: 28/104 TG3: 34/113 ^b^CG: 29/102	TG1: 3.9 (3.8) ^a^TG2: 3.4 (3.3) TG3: 4.0 (3.6) ^b^CG: 4.0 (3.9)	Hong Kong	Funded by a donation from the Simon KY Lee Fund for the Elderly in Hong Kong
Law et al. (2019) [34]	7	RCT (4-arm design)	MCI	NIAAA criteria	Not reported	TG1: 15 ^a^TG2: 16 TG3: 14 CG: 14	TG1: 76.9 (6.8) ^a^TG2: 77.9 (6.1) TG3: 71.6 (7.4) CG: 75.1 (8.5)	TG1: 7/8 ^a^TG2: 8/8 TG3: 4/10 CG: 5/9	Not reported as average years *n* (illiterate/primary/secondary/tertiary) TG1: 4/6/5/0 ^a^TG2: 5/3/7/0 TG3: 4/5/4/1 CG: 3/7/3/1	Hong Kong	Funded by the Research Grants Council (RGC) of the Hong Kong University Grants Committee
Law et al. (2022) [38]	7	RCT (4-arm design)	MCI	NIAAA criteria	Not reported	TG1: 38 ^a^TG2: 37 ^a^TG3: 34 CG: 36	TG1: 76.3 (7.2) ^a^TG2: 77.4 (6.7) ^a^TG3: 73.2 (7.3) CG: 74.1 (7.5)	TG1: 13/25 ^a^TG2: 16/21 ^a^TG3: 11/23 CG: 12/24	Not reported as average years *n* (illiterate/primary/secondary/tertiary) TG1: 8/15/13/2 ^a^TG2: 9/8/13/7 ^a^TG3: 9/12/8/5 CG: 9/14/9/4	Hong Kong	The Research Grants Council, University Grants, Committee of Hong Kong, SAR, China
Muñiz et al. (2015) [22]	8	RCT (parallel design)	MCI or probable early-stage (mild) AD	Flicker et al. criteria for MCI NINCDS-ADRDA	TG: 17.6 (0.7) CG: 17.4 (1.0)	TG: 40 CG: 40	TG: 74.9 (1.1) CG: 73.4 (1.0)	TG: 17/23 CG: 14/26	TG: 8.1 (0.6) CG: 7.3 (4.4)	Spain	None described
Nousia et al. (2018) [40]	6	RCT (parallel design)	Probable early-stage (mild) AD	NINCDS-ADRDA	Not reported	TG: 25 CG: 25	TG: 76.2 (5.14) CG: 76.3 (5.38)	TG: 9/16 CG: 5/20	TG: 8.08 (3.01) CG: 8.92 (2.83)	Greece	None described
Pantoni et al. (2017) [35]	6	RCT (parallel design)	MCI	International Working Group on MCI diagnostic criteria	TG: 27.1 (2.6) CG: 25.7 (3.2)	TG: 21 CG: 22	TG: 74.2 (6.0) CG: 75.9 (7.6)	TG: 13/8 CG: 15/7	TG: 9.0 (5.3) CG: 7.4 (3.0)	Italy	Funded by the Tuscany region and Italian Ministry of Health
Park (2022) [39]	7	RCT (parallel design	MCI	Petersen’s diagnostic criteria for MCI	MMSE Korean version TG: 26.06 (1.34) CG: 25.50 (1.31)	TG: 16 CG: 16	TG: 72.3 (5.13) CG: 70.9 (4.51)	TG: 9/7 CG: 6/10	TG: 7.56 (3.93) CG: 7.50 (2.89)	Korea	Ministry of Education of the Republic of Korea and the National Research Foundation of Korea
Rojas et al. (2013) [36]	4	RCT (parallel design)	MCI	Petersen’s diagnostic criteria for MCI CDR: 0.5	TG: 27.53 (2.33) CG: 27.13 (2.10)	TG: 15 CG: 15	TG: 72.0 (14.3) CG: 76.9 (7.1)	TG: 9/6 CG: 8/7	TG: 10.53 (3.78) CG: 10.53 (3.85)	Argentina	Co-funded by Ministry of Health of the city of Buenos Aires, Argentina, and CONICET
Rovner et al. (2018) [37]	6	RCT (parallel design)	MCI	NIAAA criteria	TG: 25.8 (2.3) CG: 25.6 (2.5)	TG: 111 CG: 110	TG: 75.5 (7.1) CG: 76.2 (6.90)	TG: 25/86 CG: 21/89	TG: 12.6 (2.2) CG: 12.4 (2.9)	USA	Funded by the National Institute on Aging
Williams et al. (2014) [42]	6	RCT (3-arm design)	MCI or mild dementia	8-item ascertain dementia score > 2	TG 1: 25.6 (2.7) ^a^TG 2: 25.7 (2.7) CG 2: 24.7 (3.1)	TG 1: 29 ^a^TG 2: 28 CG 2: 32	TG 1: 86.0 (5.9) ^a^TG 2: 83.0 (10.5) CG 2: 86.0 (4.8)	TG 1: 12/17 ^a^TG 2: 6/22 CG 2: 11/21	TG 1: 13.30 (2.6) ^a^TG 2: 15.65 (1.8) CG 2: 13.56 (2.4)	USA	Funded by the National Institute of Nursing Research and the National Institute of Deafness and Communication Disorders

*TG* treatment group, *CG* control group, *MCI* mild cognitive impairment, *AD* Alzheimer’s disease, *CDR* clinical dementia rating score, *RCT* randomized control trial, *SD* standard deviation, *DSM-IV* American Psychiatric Association, *Diagnostic and Statistical Manual of Mental Disorders*, *NIAAA* the National Institute on Aging and Alzheimer’s Association, *NINCDS-ADRDA* National Institute of Neurological and Communicative Disorders and Stroke and the Alzheimer’s disease and Related Disorders Association, *MMSE* Mini-Mental State Examination, *PEDro* Physiotherapy Evidence Database

^a^Intervention is not of interest to this review

^b^Control group is an intervention of interest

#### Intervention characteristics

Six studies implemented a CT approach [33–35, 38, 40, 41], four studies implemented a CR approach [34, 37, 39, 42], and one study implemented a CS approach [21]. Four studies used a mixed-method approach to the intervention with three studies combining CR and CT [22, 36, 41] and one study combining CS and CT [43].

Eight studies implemented a group-based approach [21, 22, 33, 34, 36, 38, 40, 43], four studies adopting a one-to-one individual approach [35, 37, 41, 42], and it was unclear which approach was adopted in the remaining study [39]. The interventions of seven studies were facilitated by an occupational therapist [34, 38, 39], a cognitive therapist [43], a research assistant who had graduated preparation and gerontological expertise [42], and a clinical psychologist [35, 36]. The remaining six studies did not indicate who administered the intervention [21, 22, 33, 37, 40, 41].

The duration of intervention sessions lasted for 45 min [41], 60 min [21, 34, 37–40, 42, 43], 120 min [33, 35, 36], and 210 min [22]. The median duration of the intervention sessions was 60 min with an average of 84 min. The frequency of the intervention sessions ranged from five sessions over a 4-month period [37] to three times per week [21, 42], with four studies having one session per week [33–35, 41], another five studies having two session per week [22, 36, 39, 40, 43], and another study having 12 sessions over an 8-week period [38]. The total duration of intervention varied from a total of 5 [37] to 1092 h [22], seven studies had up to 19 total hours of intervention [33, 34, 37–39, 41, 42], two studies had between 20 and 39 total hours of intervention [40, 43], two studies had between 40 and 49 h of intervention [35, 36], one study had 156 h of intervention [21], and one study had 1092 h of intervention [22] (Table 2).

**Table 2:** Characteristics of included studies and interventions of interest

Study	Intervention type	Intervention	Intervention delivery mode	Intervention duration	Comparison/control condition	Time of IADL data collection	IADL outcome measure	IADL outcome raw score mean (SD)	Effect for treatment group	Effect between treatment and control groups
Barban et al. (2016) [43]	Combined cognitive stimulation and cognitive rehabilitation	Reminiscence therapy in which participants created a life-story book, combined with computerized process-based cognitive training focusing on memory and executive functioning, as well as the domains of orientation, constructional praxis, abstract reasoning, and language	Group-based sessions (up to 3 participants) Facilitated by a trained cognitive therapist	2 × 60-min sessions per week over a 3-month period (total sessions: 24) Total hours: 24	Rest period	Pre intervention/baseline Post intervention 6-month follow-up	Lawton’s Instrumental Activities of Daily Living Scale [44]	Pre intervention TG: not reported CG: not reported Post intervention TG: not reported CG: not reported	Not reported for IADL	Not reported for IADL
Belleville et al. (2018) [33]	Cognitive training	Memory and attentional control strategies (e.g. visual interactive imagery, method of loci, verbal organization techniques)	Group-based sessions (4–5 participants) Facilitated	1 × 120-min session per week over an 8-week period Booster session approx. 1 week post 3-month follow-up Total hours: 16	No intervention	Pre intervention/baseline Post intervention (1 week) 3-month follow-up 6-month follow-up	The activities of daily living—prevention instrument questionnaire (ADL-PI) [45]	Pre intervention TG: 38.7 (4.95) CG: 37.7 (5.11) Post intervention TG: 40.5 (3.48) CG: 38.8 6.07) Follow-up (3 months) TG: 39.3 (4.39) CG: 38.2 (6.07) Follow-up (6 months) TG: 39.3 (4.41) CG: 38.3 (5.67)	Not reported for IADL	Not reported for IADL
Giuli et al. (2016) [41]	**MCI group:** cognitive training	**MCI group:** cognitive training for orientation, memory, categorization, and clustering	Individual sessions Facilitated Independent daily homework exercises	1 × 45-min sessions per week over a 10-week period Total hours: 7.5	No intervention	Pre intervention/baseline Post intervention	Lawton’s Instrumental Activities of Daily Living Scale [44]	**MCI group:** pre intervention TG: 7.43 (0.90) CG: 7.5 (0.80) Post intervention TG: 7.43 (0.9) CG: 7.17 (1.3)	Post intervention: no significant effect (*p* = 1.0)	Post intervention: no significant difference between the TG and CG: 0.058 (partial eta squared)
	**Probable early-stage AD group:** combined cognitive training and cognitive rehabilitation	**Probable early-stage AD group:** cognitive training programme addressing cognitive functions, including attention functions, orientation, planning of activities of daily living, and episodic and prospective memory						**Probable early-stage AD group** Pre intervention TG: 3.37 (1.9) CG: 3.37 [2] Post intervention TG: 3.63 (1.9) CG: 3.13 [2]	Post intervention: significant effect (*p* = 0.009)	Post intervention: significant difference (medium-large) between the TG and CG: 0.163 (partial eta squared
Lam et al. (2015) [21]	Cognitive stimulation	TG1: cognitive activities were leisure activities with consensus of higher demands on cognitive (e.g. reading and discussing newspapers, playing board games, calligraphy, playing a musical instrument) ^a^TG3: physical exercises including 1 × aerobic exercise session (e.g. cycling and brisk walking); 1 × mind body exercise session (e.g. Tai Chi); and 1 × stretching and toning session per week. Each session lasting 60 min	Group-based sessions Facilitated	3 × 60-min sessions per week over a 12-month period Total hours: 156	^b^CG: selection of social activities (e.g. tea gathering, film watching, shopping with friends)	Pre intervention/baseline Post intervention 8-month follow-up 12-month follow-up	Chinese Disability Assessment for Dementia Instrumental Activities of Daily Living (CDAD-IADL) [46]	Pre intervention TG1: 0.95 (0.08) ^a^TG3: 0.98 (0.05) ^b^CG: 0.95 (0.07) Post intervention (4 months) TG: 0.97 (0.06) ^a^TG3: 0.97 (0.04) ^b^CG: 0.96 (0.06) Follow-up (8 months) TG: 0.96 (0.07) ^a^TG3: 0.97 (0.05) ^b^CG: 0.98 (0.07) Follow-up (12 months) TG: 0.95 (0.07) ^a^TG3: 0.96 (0.06) ^b^CG: 0.94 (0.07)	Not reported for each individual treatment group in the study. Study included two intervention groups not of interest to this review	Not reported for each individual treatment group in the study. Study included two intervention groups not of interest to this review
Law et al. (2019) [34]	Cognitive training and cognitive rehabilitation	TG1: functional tasks exercise TG3: a computer cognitive training programme for training of attention, memory, executive function, and visual perceptual function	Group-based sessions (4–6 participants) Facilitated by an occupational therapist	12 × 60-min sessions over an 8-week period Total hours: 12	Wait-list control group	Pre intervention/baseline Post intervention	Lawton’s Instrumental Activities of Daily Living Scale [44]	Pre intervention TG 1: 20.42 (2.50) TG 3: 17.93 (5.71) CG: 18.64 (4.94) Post intervention TG 1: 22.50 (3.13) TG 3: 17.80 (6.11) CG: 18.14 (4.87)	Post intervention TG1: significant effect (*p* = 0.023) Post intervention TG3: no significant effect (*p* = 1.0)	Post intervention TG1: significant difference between the TG and CG: (mean rank = 25.97; *p* = 0.007) TG3: significant difference between the TG and CG: (mean rank = 21.18; *p* = 0.001)
Law et al. (2022) [38]		TG: a computer cognitive training programme for training of attention, memory, executive function, and visual perceptual function	Group-based sessions (4–6 participants) Facilitated by an occupational therapist	12 × 60-min sessions over an 8-week period Total hours: 12	Wait-list control group	Pre intervention/baseline Post intervention 5-month follow-up	Lawton’s Instrumental Activities of Daily Living Scale [44]	Pre intervention TG: 18.76 (5.11) CG: 18.44 (4.16) Post intervention TG: 18.76 (5.29) CG: 17.64 (4.73) Follow-up (5 months) TG: 18.26 (5.31) CG: 17.61 (4.36)	Adjusted mean difference (post intervention) compared with control (95% *CI*): 1.32 (−0.24–2.87) (*p* = 0.000)	Post intervention: the TG group did not show any significant between-group differences in any outcomes compared to the CG
Muñiz et al. (2015) [22]	Combined cognitive training and cognitive rehabilitation	Cognitive and motor stimulation interventions combined with activity of daily living training	Group-based sessions (5–7 participants) Facilitated	2 × 210-min sessions per week over a 3-year period Total hours: 1092	Written materials about AD and were invited to make use of a helpline by calling the study secretary (an experienced social worker)	Post intervention (1 month) During intervention: 3, 6, 12, 18, and 24 months Post intervention (36 months)	Functional Activities Questionnaire [47]	Pre intervention TG: not reported CG: not reported During intervention (1 month) TG: −0.84 (0.85) CG: −0.09 (0.77) During intervention (3 months) TG: 1.91 (0.82) CG: 1.37 (0.84) During intervention (6 months) TG: 2.58 (0.83) CG: 4.47 (0.96) During intervention (12 months) TG: 4.58 (0.89) CG: 6.72 (0.95) During intervention (18 months) TG: 6.34 (1.10) CG: 8.38 (1.37) During intervention (24 months) TG: 8.47 (1.14) CG: 10.90 (1.12) Post intervention: (36 months) TG: 13.74 (1.23) CG: 13.19 (1.44)	The effect for IADL was reported at the 24-month analysis only and not at the end of the intervention period The study by group regression coefficient represents the monthly cumulative difference between the TG and the CG in the dependent variable; the obtained results correspond to a difference of 2.64 (95% *CI* 0.48 to 4.8) in IADL at the 24-month period of intervention	More rapid deterioration is observed in the control group (*p* = < 0.05)
Nousia et al. (2018) [40]	Cognitive training	RehaCom software package — cognitive training in several domains with an emphasis on episodic and delayed memory, attention, processing speed, and executive functions and language exercises utilizing pen and paper supplemented by cognitive-linguistic exercises for homework. The language intervention consisted exercises of morphology, syntax, semantics, naming, verbal fluency, and word recall	Group-based sessions Facilitated Independent daily homework exercises	2 × 60-min sessions over a 15-week period Total hours: 30	Wait-list control group	Pre intervention/baseline Post intervention	Instrumental Activities of Daily Living (IADL) questionnaire	Pre intervention TG: 13.60 (2.10) CG: not reported Post intervention TG: 12.64 (1.57) CG: not reported	Not reported for IADL	Not reported for IADL
Pantoni et al. (2017) [35]	Cognitive training	Attention Process Training-II consisting of a group of hierarchically organized tasks aimed at exercising different components of attention (focused, sustained, selective, alternating, and divided)	Individual sessions Facilitated by a clinical neuropsychologist	1 × 120-min sessions per week over a 20-week period Total hours: 40	Participants were instructed to have a usual lifestyle and were provided of medication and clinic consultations as usually needed	Pre intervention/baseline Post intervention (6 months) 12-month follow-up	Lawton’s Instrumental Activities of Daily Living Scale [44]	Pre intervention TG: 1.90 (2.07) CG: 2.23 (2.39) Post intervention (6 months) TG: 2.29 (2.55) CG: 2.23 (2.41) Follow-up (12 months) TG: 2.57 (2.80) CG: 3.23 (2.59)	Not reported	No significant difference between the TG and CG: *p* = 0.457
Park (2022) [39]		Cognitive rehabilitation: virtual shopping training using the virtual supermarket application	Unclear if individual or group-based sessions Facilitated by an occupational therapist	2 × 60-min sessions per week over an 8-week period Total hours: 16	Wait-list control group	Pre intervention/baseline Post intervention	Korean Instrumental Activities of Daily Living [48]	Pre intervention TG: 16.69 (3.86) CG: 17.81 (2.68) Post intervention TG: 19.63 (3.53) CG: 18.06 (2.88)	Within-group changes (TG) 2.93 (2.56) *p* = < 0.001	Between-group (TG and CG) differences (95% *CI*): 2.688 (1.31; 4.05) *p* = < 0.01
Rojas et el. (2013) [36]	Combined cognitive training and cognitive rehabilitation	Cognitive stimulation training utilizing episodic memory encoding strategies and involving group activities based on cognition and social functioning. Instructions on the use of strategies and external aids (checklists, calendars, etc.) to support cognitive functioning were also provided	Group-based sessions Facilitated by a clinical neuropsychologist	2 × 120-minute sessions per week over a 6-month period Total hours: 48	No intervention	Pre intervention/baseline Post intervention	Lawton’s Instrumental Activities of Daily Living Scale [44]	Pre intervention TG: 1.00 (1.81) CG: 0.73 (2.31) Post intervention TG: 0.43 (0.85) CG: 0.92 (1.38)	Post intervention: no significant effect	Not reported for IADL
Rovner et al. (2018) [37]	Cognitive rehabilitation	Behavioural activation using goal setting and action plans. Action plans relied on visual cues, written schedules, step-by-step sequencing, and procedural memory to compensate for cognitive deficits	Individual sessions Facilitated	5 × 60-min sessions over a 4-month period and 6 × 60 minute follow-up maintenance sessions over the 20-month period Total hours: 5 + 6	Discussions focused on the experience of ageing, memory loss, illness, disability, and social isolation	Pre intervention/baseline Post intervention (6 month) 12-month follow-up 18-month follow-up 24-month follow-up	The University of California Performance-Based Skills Assessment [49]	Pre intervention TG: 71.51 (13.33) CG: 71.60 (11.59) Post intervention: (6 months) TG: 69.07 (15.62) CG: 70.32 (15.10) Follow-up (12 months) TG: 71.43 (18.01) CG: 69.43 (16.88) Follow-up (18 months) TG: 71.20 (15.91) CG: 67.54 (16.70) Follow-up (24 months) TG: 73.36 (15.85) CG: 67.51 (19.01)	TG had stable University of California Performance-Based Skills Assessment (function) scores over time (slope, −0.13; 95% *CI* [−2.05 to 1.79]; *p* = 0.89)	The CG participants declined (−2.60; 95% *CI* [−4.36 to −0.83]; *p* = .004) The difference in slopes was 2.47 (95% *CI*, −0.14 to 5.07; *p* = .06). The mixed-effects analysis of all randomized participants produced a similar result (difference in slopes, 2.71; 95%, *CI* [0.12 to 5.30]; *p* = .04)
Williams et al. (2014) [42]	Cognitive rehabilitation	TG 1: Training in problem-solving and inductive reasoning strategies for everyday activities with a focus on scheduling activities, medications, eating out, and nutrition choices	Individual sessions Facilitated by a trained research assistant	6 × 60-min sessions over a 3-week period Total hours: 6	No intervention	Pre intervention/baseline Post intervention 3-month follow-up 6-month follow-up	Everyday problems test for cognitively challenged elders [50]	Pre-intervention TG 1: 12.69 (7.6) CG: 13.34 (8.1) Post intervention TG 1: 3.10 (5.2) CG: −0.72 (6.8) Follow-up (3 months) TG 1: 2.71 (5.4) CG: −1.15 (6.3) Follow-up (6 months) TG 1: 1.67 (5.6) CG: 0.92 (5.5)	Post intervention: significant effect (*p* = 0.01)	Significant difference between the TG and CG (*d* = 3.82; *p* = 0.01)

*IADL* instrumental activity of daily living, *SD* standard deviation, *TG* treatment group, *CG* control group, *MCI* mild cognitive impairment, *AD* Alzheimer’s disease

^a^Intervention is not of interest to this review — this intervention group will be considered the ‘control group’ for the meta-analysis

^b^Control group is an intervention of interest — this control group will be considered as a ‘treatment group’ for the meta-analysis

#### Methodological characteristics

##### Study design

All studies are RCTs, with three studies using an active control [21, 22, 37] and ten using inactive control condition [33–36, 38–43].

##### Number of participants

Sample sizes for studies ranged between 30 participants [36] and 555 participants [21] with a median of 85 participants.

##### IADL outcome measure

Six studies [34–36, 38, 41, 43] measured IADL performance with the Lawton’s Instrumental Activities of Daily Living scale [44]. The other seven studies [21, 22, 33, 37, 39, 40, 42] used other measures: the University of California Performance-Based Skills Assessment [49], Every Day Problems Test for Cognitively Challenged Elders [50], Functional Activities Questionnaire [47], Chinese Disability Assessment for Dementia — Instrumental Activities of Daily Living [46], Korean Instrumental Activities of Daily Living Scale [48], and Activities of Daily Living—Prevention Instrument questionnaire [45].

##### Study duration

Study duration ranged from 3 to 10 weeks [33, 34, 38, 39, 41, 42], 15 to 20 weeks [35, 40], 3 to 4 months [37, 43], 6 to 12 months [21, 36], and 3 years [22].

##### Duration of follow-up

Seven studies [21, 33, 35, 37, 38, 42, 43] had follow-up periods following post-intervention data collection. There was a considerable variation with follow-up period duration across these seven studies; two studies had a 3-month follow-up [33, 42], one study had a 4-month follow-up [21], one study had a 5-month follow-up [38], four studies had a 6-month follow-up [33, 35, 37, 42], one study had an 8-month follow-up [21], one study had a 12-month follow-up [37], and one study had an 18-month follow-up [37].

##### Study origin

Two studies were conducted in the USA [37, 42]; three in Hong Kong [21, 34, 38]; two in Italy [35, 41]; one study conducted in Canada [33], Korea [39], Spain [22], Greece [40], and Argentina [36]; and the final study was conducted across four countries: Italy, Greece, Norway, and Spain [43].

#### Risk of bias

PEDro scores of the included studies ranged from 4/10 to 8/10 (Supplementary Material 2). Twelve out of the 13 included studies were considered to have a low risk of bias [21, 22, 33–35, 37–43] with one study indicating moderate risk of bias [36].

A common area of bias was non-blinding of the participants (criterion 5) or therapists (criterion 6) during the intervention; all studies included in this review failed to address at least one of the two criteria. In studies, the assessor for IADL performance was not blinded to which group the participant had been allocated [36, 40, 41, 43]. Only four studies reported concealment allocation [22, 39, 41, 43].

##### Outcome of intervention

Three studies found no statistically significant evidence for improving IADL performance [36–38], whilst two studies found a significant positive effect [39, 42]. Giuli et al. [41] found CR combined with CT to have statistically significant evidence for improving IADL performance among participants with early-stage dementia but found insufficient statistical evidence to conclude improvements for participants with MCI. Law, Mok [34] found CR to be statistically significant for improving IADL performance but CT not to be significantly effective. Although the remaining six studies administered an IADL outcome measure, they did not report the effectiveness of the intervention on IADL performance [21, 22, 33, 35, 40, 43] (Table 2).

### Results of meta-analysis

The meta-analysis included eleven studies with a total of 1167 participants assessing the immediate effect of cognitive remediation on IADL performance. Six studies reported follow-up data and were included in the meta-analysis of long-term carryover effects. Of these, three studies reported data at 3–5 months [21, 33, 38, 42], four studies reported data at 6–8 months [21, 33, 35, 37, 42], and one study reported data at 12 months [37] post-intervention.

**Fig. 2.**
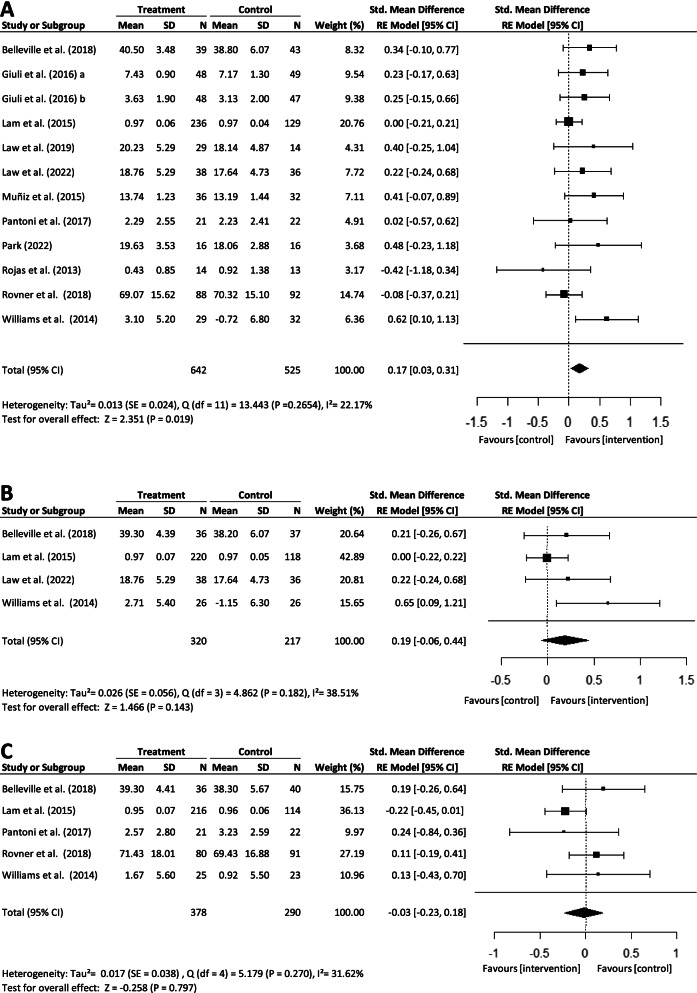
Forest plot of the effect of cognitive remediation on IADL performance compared to control at **A** immediate post-intervention from nine studies, **B** 3–5 months post-intervention from three studies, and **C** 6–8 months post-intervention from five studies

The immediate post-intervention results of cognitive remediation indicated that IADL performance was superior in the intervention group when compared with the control group (*SMD*: 0.17, 95% *CI*: 0.03 to 0.31), with small effect size (*Z* = 2.35, *P* = < 0.02) (Fig. 2A). The *I*^2^ statistics indicated heterogeneity might not be important [26] (*I*^2^ = 22.17%, *df* = 11, *P* = 0.27). There was little statistical evidence of publication bias (Supplementary material 5). The largest two studies returned null findings with positive findings restricted to smaller studies. However, two smaller studies with null or negative findings contradict this possible pattern. The Begg and Egger tests were not statistically significant with *p*-value = 0.076 and *p*-value = 0.250, respectively. When separated into subgroups, there was insufficient statistical evidence for carryover effect at 3–5 months or 6–8 months (Fig. 2B–C).

### Results in subgroup analysis

#### Type of intervention

**Fig. 3:**
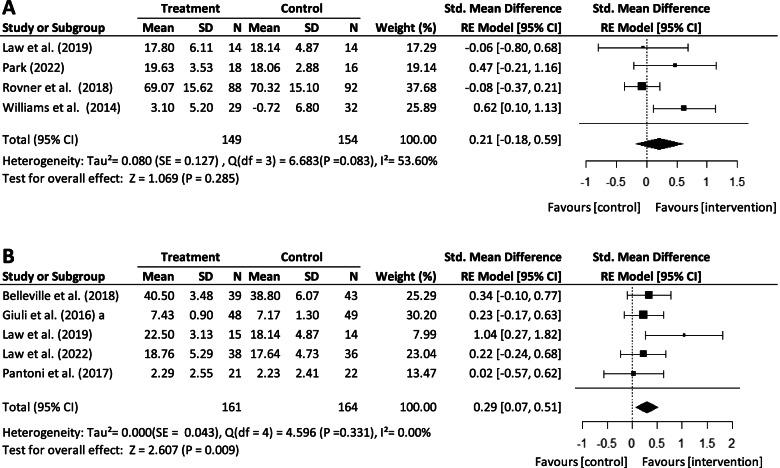
Forest plot of the effect of cognitive remediation approaches on IADL performance compared to control. **A** Cognitive rehabilitation. **B** Cognitive training

When compared to control group outcomes, studies using a CT approach [33–35, 38, 41] had a significant but overall small effect on IADL performance (*SMD*: 0.29; 95% *CI*: 0.07 to 0.51. Effect size *Z* = 2.607, *p* = 0.01). No significant differences were found between groups in studies using a CR approach [34, 37, 39, 42] (*SMD*: 0.21; 95% *CI*: −0.18 to 0.59) (Fig. 3A–B).

#### Duration of intervention

Interventions less than 10 h in total [41, 42] appeared to have the largest effect size (*SMD*: 0.33; 95% *CI*: 0.08 to 0.58); however, the overall effect size was small (*Z* = 2.6032, *p* = 0.01). This was followed by interventions lasting between 10 and 20 h [33, 34, 37–39] (*SMD*: 0.19; 95% *CI*: −0.06 to 0.43) and interventions lasting between 21 and 50 h [35, 36] (*SMD*: −0.14; 95% *CI*: −0.62 to 0.33) (Supplementary Material 3).

#### Individual vs group-based interventions

Four studies included individual intervention sessions [35, 37, 41, 42] and six with group-based intervention sessions [21, 22, 33, 34, 36]. The SMDs were almost identical between groups, but the smaller sample sizes in the subgroup analyses provided insufficient statistical evidence of the therapeutic benefit of either approach (individual intervention: *SMD*: 0.18; 95% *CI*: −0.06 to 0.41; *Z* = 1.47; *p* = 0.14; group-based intervention: *SMD*: 0.14; 95% *CI*: −0.10 to 0.37; *Z* = 1.13; *p* = 0.26) (Supplementary Material 4).

### Evaluating the quality of evidence

The quality of evidence was evaluated via GRADEpro. The quality of evidence was determined to be moderate for the outcome of IADL performance. The true effect size is likely to be close to the effect estimate reported in this meta-analysis; however, there is a possibility that it is substantially different.

## Discussion and implications

### Main findings

The present study is the first to analyse the effects of cognitive remediation on IADL performance in older adults with MCI and early-stage dementia. Based on results from nine RCTs, cognitive remediation improved IADL performance immediately post-intervention with a small overall effect in older adults with MCI and early-stage dementia. However, with smaller sample sizes, there was insufficient statistical evidence to confirm a longer-term effect.

Of the three types of cognitive remediation approaches included in this review, CT was shown to have a greater effect size when compared to control than CR compared to control and CS compared to control. CT refers to the restorative strategy to improve cognitive functioning through repeated practice on theoretically driven activities targeting specific cognitive domains [51]. A decline in cognition associated with MCI and early-stage dementia has been shown to affect performance in daily activities [52, 53]. Therefore, by targeting the cognitive domains that are required to carry out IADL, it is expected improvements in these cognitive domains are transferable to IADL performance. The effects of CT on IADL performance can be explained by previous research that shows the ability to perform IADL is dependent on intact cognition, particularly executive functioning [54, 55], and that improvements in cognition, particularly executive functioning, is associated with improved IADL performance [7, 10, 13].

We postulate that CR should be individualized and tailored to the individual’s needs. Four studies [34, 37, 39, 42] were included in this sub-analysis, in which one study [34] employed a group-based rather than an individualized approach. Difficulties in daily life can be relatively different between each participant. It is uncertain if a group-based format can provide interventions to match the individual’s needs. This might be a possible reason for an overall insignificant finding of CR. Further to this, the benefits associated with CR are specific to the individual practiced activities and may not transfer to IADL [56–58]. It is unclear, and it is unlikely, the tasks practised in these studies represented those assessed in the IADL outcomes used. The effect may not generalize.

In previous studies, general CS including recreational activities and social groups have shown to improve general cognitive functioning [11, 59]; however, these studies did not look into IADL performance. Furthermore, these general CS activities may not improve specific cognitive abilities [59, 60]. These could be the reasons to explain the insignificant findings of this current review. IADL performance among older adults has been shown to be reliant on the specific cognitive domains of praxis/visuospatial skills [61] and executive functioning [61, 62]. Further to this, CS aims to enhance general cognitive and social functioning [63], the IADL outcome measures utilized in the studies and included in this review did not thoroughly address activities around communication management and community engagement.

It is worth noting that in this review, effect sizes were relatively small, and hence, the sample size required to establish statistical significance is quite large. There is yet, insufficient evidence to confirm the effects of CR and CS, suggesting further research may be warranted to determine if these small effect sizes are of clinical interest. Further to this, there was only one study in the meta-analysis for CS, whilst there were three for CR and four for CT. This also potentially influencing the results as to why CT showed significant differences with the control, but not CR or CS.

Both individualized and group-based cognitive remediation showed similar clinical effects, although these effects were too small to be detected as statistically significant on the available evidence. This finding is consistent with a previous meta-analysis that showed nonsignificant findings between individual and group cognitive remediation (CT and CR) in individuals with Alzheimer’s disease [64]. It must be acknowledged that CT is rarely individualized, and it has limited capacity to be modified according to an individual’s needs and coping strategies. CR, however, eliminates these factors as it focuses on providing an individualized program according to an individual’s deficits and functional goals [65, 66]. The fact that CT is difficult to administer due to its individualized nature and focus on functional goals [67], as well as relatively modest effect sizes, cost-benefit analyses may be warranted to test whether the intervention is worth pursuing further.

The interventions with the shortest duration (less than 10 h) showed the greatest effect when compared to control. The results are consistent with a previous systematic review which reported intervention periods of 6 to 20 h to be the most effective in enhancing memory, quality of life, and mood for older adults with MCI [67]. Considering people with MCI and dementia frequently display reduced ability to maintain attention, shorter intervention sessions may be more favourable. Further to this, MCI and dementia are known to be degenerative in nature, and a decline in cognition over time is expected. The two studies in this sub-analysis had interventions lasting at least 12 months; therefore, it is possible that further cognitive decline occurred during this time and consequently limited the findings of effectiveness regarding IADL performance. However, it must be noted that although the shortest duration showed the greatest effect in this review, extrapolating this back to the wider population is not supported. There is insufficient statistical evidence to conclude that duration has this effect in the wider population.

### Validity of observations and limitations

#### Source of bias

Although 12 out of the 13 included studies were considered to have a low risk of bias [21, 22, 33–35, 37–43], inadequate participant, and therapist blinding, concealment of allocation was an issue in most studies. The maximum PEDro score is 11; realistically, the maximum achievable score for this type of trial is 9 due to challenges in cognitive remediation trials in blinding participants (criterion 5) and therapist (criterion 6). All studies included in this review did not fulfil criteria 5 or 6 [21, 22, 33–43]. Lack of blinding introduces expectation bias and potentially overstated results. Further to this, 10 of the 13 studies failed to report concealment allocation (criterion 3), which potentially introduces systematic biases in random allocation [21, 33–38, 40, 42]. Evidence suggests an association between concealment and effect size [68].

#### Limitations

This review had several limitations. Firstly, included studies utilized many different measurement instruments, making it difficult to compare findings. In addition, although studies reported IADL performance as an outcome, this was usually secondary to other outcomes such as cognitive functioning. Further to this, instruments used to measure IADL performance, such as the Lawton’s IADL scale, have been shown to have a ceiling effect when used in a population of individuals with dementia [69]. Secondly, all sub-analyses included small number of studies that limited group comparisons. Furthermore, a lack of follow-up data makes it difficult to draw conclusions regarding long-term carryover effects or impact on IADL performance. Thirdly, studies using cognitive remediation as both treatment and control were excluded from this review. Finally, studies that did not use strict diagnostic criteria for MCI were excluded to reduce heterogeneity often found between participants in MCI studies. Additionally, this review did not differentiate between amnestic MCI and non-amnestic MCI and included participants with either MCI or early-stage dementia. Due to the complex and varied nature of these diagnosis, there may be differences in the effectiveness of cognitive remediation between participants that were not evaluated in this review, reducing the generalizability of the results. Whilst this review synthesizes existing literature and the risk of bias was low, the limited number of studies, small sample sizes, heterogeneity of diagnosis, interventions, and outcome measures indicates that some caution is required when considering the results of this systematic review and meta-analysis.

#### Implications for research

One consistent observation is that the clinical effect is relatively small when considering a general rule of thumb reported by Cohen, in which a SMD of 0.2 represents a small effect, an SMD of 0.5 represents a medium effect, and an SMD of 0.8 represents a large effect [70]. All but four of the studies reported effects between 0 and 0.4 SMD with the combined estimate 0.17 SMD. This systematic review found insufficient evidence to support the use of specific cognitive remediation approaches in clinical practice to improve IADL performance; however, this may reflect a lack of high-quality RCTs in the field. There is a need for large RCTs to have sufficient power to identify functional improvements in IADL performance.

The standardization of outcome measures between RCTs is also suggested as it would avoid problems associated with heterogeneity and risk of bias. It is also recommended that a network meta-analysis is conducted to provide an answer for comparing the effectiveness of the three cognitive remediation approaches. Further studies are required to determine what cognitive remediation approaches are best for individuals with MCI in comparison with those with dementia.

## Conclusion

Given the impact that cognitive impairment associated with MCI and early-stage dementia has on IADL performance, the need for intervention is clear. This review reveals that cognitive remediation has significant immediate positive effects on IADL performance, but there is insufficient statistical evidence to confirm any lasting effect. Whilst results are promising, due to the small number of RCTs and small sample sizes, firm conclusions about the effectiveness of the three types of cognitive remediation cannot be drawn. More studies with larger sample sizes and follow-up periods are needed to inform immediate and long-term effectiveness of cognitive remediation on IADL performance.

## Supplementary Information

**Additional file 1: Supplementary Material 1.** Search strategy conducted in Medline. **Supplementary Material 2.** Risk of bias summary: Review authors’ judgements about each risk of bias item for each included study. **Supplementary Material 3.** Forest plot of the effect of cognitive interventions on IADL performance compared to control (A) less than 10 hours of intervention, (B) 10-20 hours of intervention, and C) 21-50 hours of intervention. **Supplementary Material 4.** Forest plot of the effect of cognitive interventions on IADL performance compared to control (A) group intervention, and (B) individual intervention. **Supplementary Material 5.** Funnel plot for publication bias of effect of cognitive remediation on IADL performance among older adults with MCI and early-stage dementia.

## Data Availability

The datasets used and/or analysed during the current study are available from the corresponding author on reasonable request.
