# Supplementary material for: Efficacy of cognitive remediation on activities of daily living in individuals with mild cognitive impairment or early-stage dementia: a systematic review and meta-analysis
Source: Syst Rev. 2022 Aug 2;11:156. doi: 10.1186/s13643-022-02032-0 (PMC9344685; doi:10.1186/s13643-022-02032-0)

**Supplementary Material 1**. Search strategy conducted in Medline

Database: Ovid MEDLINE(R) ALL

Search Strategy:

--------------------------------------------------------------------------------

1 exp Dementia/ or exp Cognitive Dysfunction/ or exp Alzheimer Disease/ or exp Cognition Disorders/ (215962)

2 ((mild adj dementia) or MCI or dementia).tw. (110900)

3 ((cognitive adj2 dementia) or CIND).tw. (2361)

4 memory disorders/ or exp amnesia/ (28077)

5 ((age-associated adj2 impairment) or AAMI).tw. (790)

6 ((age-related adj2 impairment) or (memory adj impairment) or (memory adj decline) or (memory adj loss) or

(impaired adj memory)).tw. (16840)

7 (alzheimers or (cognitive adj decline) or (memory adj decline)).tw. (131060)

8 1 or 2 or 3 or 4 or 5 or 6 or 7 (323146)

9 exp Cognitive Therapy/ or (cognitive adj therap$).tw. (27399)

10 (cognitive and (intervention or training or techniques or restoration or retraining or re-training or stimulation

or rehabilitation or remediation)).tw. (61294)

11 exp Neurological Rehabilitation/ or Rehabilitation/ (30345)

12 exp Mental Recall/ or (mental adj stimulation).tw. (32832)

13 (task adj2 training).tw. (1684)

14 exp Occupational Therapy/ or (occupational adj rehabilitation).tw. (13269)

15 ((sensory adj stimulation) or (reminiscence adj therapy)).tw. (2754)

16 exp "Imagery (Psychotherapy)"/ or (mental adj imagery).tw. (3004)

17 (skill and (acquisition or retention)).tw. (4734)

18 exp Learning/ or (memory adj training).tw. (369132)

19 (memory and (encoding or retrieval)).tw. (19763)

20 ((task adj2 training) or (functional adj task)).tw. (2072)

21 ((guided adj imagery) or (motor adj imagery)).tw. (3124)

22 Visual Perception/ or Cues/ (91246)

23 (visualisation or cues).tw. (71500)

24 9 or 10 or 11 or 12 or 13 or 14 or 15 or 16 or 17 or 18 or 19 or 20 or 21 or 22 or 23 (580668)

25 exp "Activities of Daily Living"/ or ADL.tw. (100797)

26 ((Instrumental adj3 living) or (activities adj living) or IADL).tw. (3190)

27 (functional and (performance or ability or status)).tw. (178208)

28 (daily and (task or activities)).tw. (62300)

29 ((complex adj activities) or (task adj performance) or (day adj2 activities)).tw. (12258)

30 25 or 26 or 27 or 28 or 29 (317962)

31 8 and 24 and 30 (5810)

32 exp randomized controlled trial/ or random$.tw. (1189300)

33 randomized controlled trial.pt. (487802)

34 32 or 33 (1189300)

35 31 and 34 (988)

36 exp "Aged, 80 and over"/ or exp Aged/ or exp Middle Aged/ (4843004)

37 (older or elder$ or aged).tw,kf. (1015920)

38 36 or 37 (5310183)

39 35 and 38 (765)

40 limit 39 to (english language and yr="2009 -Current") (571)

**Supplementary Material 2**. Risk of bias summary: Review authors’ judgements about each risk of bias item for each included study.

| **Physiotherapy Evidence Database (PEDro)** | | | | | | | | | | | | | |
| --- | --- | --- | --- | --- | --- | --- | --- | --- | --- | --- | --- | --- | --- |
| Study | Criterion 1  Eligibility Criteria | Criterion 2  Random Allocation | Criterion  3  Allocation Concealment | Criterion 4  Similarity of Baseline Measures | Criterion 5  Subject Blinding | Criterion 6  Therapist Blinding | Criterion 7  Assessor Blinding | Criterion 8  Adequate Follow-up | Criterion 9  Intention to Treat Analysis | Criterion  10  Between-group Comparisons | Criterion 11  Point estimates and variability | Total Score  (Max. 10) | Low/ Mod/ High Risk |
| Barban et al. (2016) (1) | YES | YES | YES | YES | NO | NO | NO | YES | NO | YES | YES | 6 | Low |
| Belleville et al. (2018) (2) | YES | YES | NO | YES | NO | NO | YES | YES | YES | YES | YES | 7 | Low |
| Giuli et al. (2016) (3) | YES | YES | YES | YES | NO | NO | NO | YES | NO | YES | YES | 6 | Low |
| Lam et al. (2015) (4) | YES | YES | NO | YES | NO | NO | YES | NO | YES | YES | YES | 6 | Low |
| Law et al. (2019) (5) | YES | YES | NO | YES | NO | NO | YES | YES | YES | YES | YES | 7 | Low |
| Law et al. (2022) (6) | YES | YES | NO | YES | NO | NO | YES | YES | YES | YES | YES | 7 | Low |
| Muniz et al. (2015) (7) | YES | YES | YES | YES | NO | NO | YES | YES | YES | YES | YES | 8 | Low |
| Nousia et al. (2018) (8) | YES | YES | NO | YES | NO | NO | NO | YES | YES | YES | YES | 6 | Low |
| Pantoni et al. (2017) (9) | YES | YES | NO | YES | NO | NO | YES | YES | NO | YES | YES | 6 | Low |
| Park (2022) (10) | YES | YES | YES | YES | NO | NO | YES | YES | NO | YES | YES | 7 | Low |
| Rojas et el. (2013) (11) | YES | YES | NO | YES | NO | NO | NO | NO | NO | YES | YES | 4 | Mod |
| Rovner et al. (2018) (12) | YES | YES | NO | YES | NO | NO | YES | NO | YES | YES | YES | 6 | Low |
| Williams et al. (2014) (13) | YES | YES | NO | YES | NO | NO | YES | YES | NO | YES | YES | 6 | Low |

**Supplementary Material 3**. Forest plot of the effect of cognitive interventions on IADL performance compared to control (A) less than 10 hours of intervention, (B) 10-20 hours of intervention, and C) 21-50 hours of intervention.

**Supplementary Material 4**. Forest plot of the effect of cognitive interventions on IADL performance compared to control (A) group intervention, and (B) individual intervention.

**Supplementary Material 5.** Funnel plot for publication bias of effect of cognitive remediation on IADL performance among older adults with MCI and early-stage dementia.
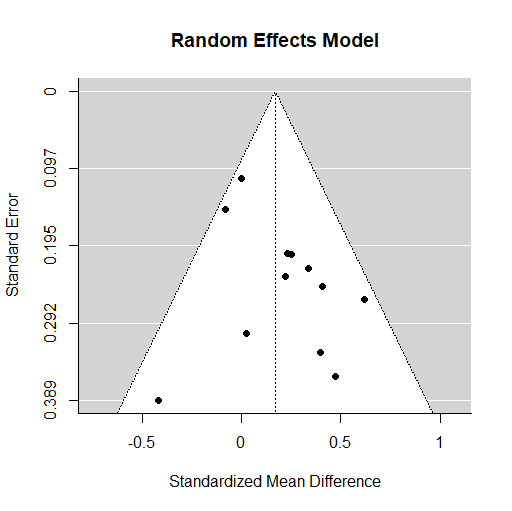

Supplement: Supplementary file 1 — Additional file 1: Supplementary Material 1. Search strategy conducted in Medline. Supplementary Material 2. Risk of bias summary: Review authors’ judgements about each risk of bias item for each included study. Supplementary Material 3. Forest plot of the effect of cognitive interventions on IADL performance compared to control (A) less than 10 hours of intervention, (B) 10-20 hours of intervention, and C) 21-50 hours of intervention. Supplementary Material 4. Forest plot of the effect of cognitive interventions on IADL performance compared to control (A) group intervention, and (B) individual intervention. Supplementary Material 5. Funnel plot for publication bias of effect of cognitive remediation on IADL performance among older adults with MCI and early-stage dementia. [file 13643_2022_2032_MOESM1_ESM.docx]
